# Pressure pain thresholds over the cranio-cervical region in headache: a systematic review and meta-analysis

**DOI:** 10.1186/s10194-018-0833-7

**Published:** 2018-01-26

**Authors:** René F. Castien, Johannes C. van der Wouden, Willem De Hertogh

**Affiliations:** 1 Department of General Practice and Elderly Care Medicine, Amsterdam Public Health research institute, VU University Medical Center, van der Boechorststraat 7, Amsterdam, 1081 BT the Netherlands; 2 Healthcare center Haarlemmermeer, Waddenweg, Hoofddorp, 2134 XL the Netherlands; 3 Department of Rehabilitation Sciences and Physiotherapy, Faculty of Medicine and Health Sciences, University of Antwerp, Campus Drie Eiken, D.S.022, Universiteitsplein 1, 2610 Wilrijk, Belgium

**Keywords:** Algometry, Trapezius, Headache, Sensitization, Case-control

## Abstract

Sensitivity of tissues can be measured by algometry. Decreased pressure pain thresholds over the cranio-cervical area are supposed to reflect signs of sensitization of the trigemino-cervical nucleus caudalis. A systematic review was conducted to assess the current scientific literature describing pressure pain threshold (PPT) values over the cranio-cervical region in patients with migraine, tension-type headache (TTH), and cervicogenic headache (CeH). A literature search was executed in three databases. The search strategy included the following keywords: migraine, TTH, CeH, PPT and algometry. A total of 624 papers was identified of which relevant papers were subsequently assessed for methodological quality. Twenty-two selected papers were assessed by two independent reviewers and the majority of studies scored low risk of bias on the selected items. Mean PPT values of several sites measured in the cranio-cervical region in patients with migraine, chronic TTH and CeH scored lower values compared to controls. The trapezius muscle (midpoint between vertebrae C7 and acromion) was the most frequently targeted site and showed significantly lower PPT values in adults with migraine (pooled standardized mean difference kPa: 1.26 [95%CI -1.71, −0.81]) and chronic TTH (pooled standardized mean difference kPa: -2.00 [95%CI -2.93, −1.08]). Most studies found no association between PPT values and headache characteristics such as frequency, duration or intensity. Further standardization of PPT measurement in the cranio-cervical region is recommended.

**Electronic supplementary material:**

The online version of this article (10.1186/s10194-018-0833-7) contains supplementary material, which is available to authorized users.

## Review

### Background

Pressure pain thresholds (PPT) reflect sensitivity and can be measured by pressure algometry using a mechanical or electronic pressure algometer. Pressure is gradually increased and subjects have to report when the applied pressure changes from a feeling of pressure into a feeling of pressure and pain. Pressure algometry has been shown to be a valid and reliable measurement of PPT in cranio-cervical muscles [1–3]. Pain perception studies in headache patients measuring muscle tenderness, including PPT, have clarified the pathophysiological mechanism in different types of headache. PPT represent the sensitivity of tissues and depending on the site of measurement (cervico-cephalic and/or extra-cervico-cephalic region) where these PPT are decreased, they are supposed to reflect signs of sensitization of the trigemino-cervical nucleus caudalis [4–6]. This neurophysiological model of sensitization of the trigemino-cervical nucleus caudalis is generally presumed to play an important role in the onset and maintenance of chronic headaches including migraine and chronic tension-type headache (TTH) [7, 8]. Consequently, people with headache can be expected to have lower PPT values in the cranio-cervical region.

A narrative review reported on studies describing the correlation between PPT in the cervico-cephalic region and different types of headache in the period till 2010 [4]. The majority of studies included in that review showed several methodological shortcomings in not fulfilling the ICHD II criteria and standardization of PPT measurements. Recently, a systematic review described the correlation between headache and PPT in muscles in the trigeminal areas [9]. However, to date, no aggregated evidence on the association between PPT values in the cranio-cervical region and headache is available. This can be of interest, given the ongoing development of therapeutic interventions in the cranio-cervical region.

Currently, non-pharmacological interventions to treat headaches are widely administered as a prophylactic treatment option for migraine, TTH or cervicogenic headache (CeH). These include physical- and manual therapy, neuromodulation by botox toxine injections and greater occipital nerve or cervical joint anesthesia [10, 11]. Physical- and manual therapy interventions are predominantly directed to the cranio-cervical region in order to reduce headaches [11]. The rationale for administering these interventions is that effecting a decrease of afferent nociceptive information in the cranio-cervical region (i.e. a peripheral mechanism) will lead to a decrease of peripheral sensitization or sensitization of the trigemino-cervical nucleus caudalis [12, 13]. Therefore, providing clinicians who administer such interventions with reference PPT values in the cranio-cervical region may assist them in their evaluation of PPT values.

The question of this review is whether PPT values in the cranio-cervical region in participants with migraine, TTH and CeH are decreased compared to healthy controls. Additionally, it will be of further interest to assess the strength of association between PPT values and headache characteristics such as frequency, duration or intensity.

## Methods

### Identification and selection of studies

Based on our research question a systematic, computer-based literature search was conducted by an independent librarian (HdK) employed by the VU University in August 2015 in literature published during the period January 2004–August 2015. This limitation of the search period was to ensure the inclusion of studies that were published after the publication of the updated and more detailed classification of headaches, especially TTH (ICHD II, 2004). The medical databases included in the search were: PubMed, Cinahl, and Embase. The following words were used to search in all databases: headache, pressure pain threshold and algometry (see Additional file 1: Appendix 1 for full search strategy). We did not perform an additional search for grey literature.

Two reviewers (RC, WDH) independently screened the titles and abstracts of the citations generated by the literature search. The following inclusion criteria were applied to decide if papers would be included for further evaluation: (a) headaches were classified as migraine, TTH or CeH, (b) pain threshold measurements (algometry) were applied in the cranio-cervical region, (c) scores on PPT were available, (d) research involved humans, (e) were case-control studies, and (f) research was published after 2004. Case studies were excluded.

**Fig. 1:**
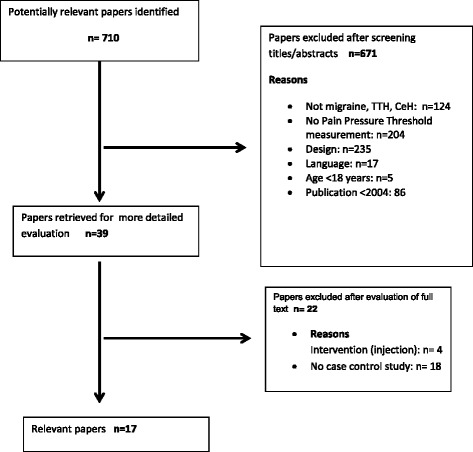
Flow chart of study selection

The search generated 868 papers. After manual removal of duplications 710 papers were subsequently screened for eligibility for title and abstract. Finally, based on full-texts screening 17 articles out of 39 papers met the in- and exclusion criteria of our search and were carefully analyzed. More detailed information of this procedure is provided in the flow chart (Fig. 1).

### Assessment of characteristics of studies

To evaluate the methodological quality of the included studies the Dutch EBRO checklist for case-control studies was used, as all included studies had this design (http://netherlands.cochrane.org). The five most relevant checklist items regarding selection bias, blinding and determination of confounders were used to assess risk of bias in the included studies. These five selected criteria items were, independently, scored by two reviewers (RC, WD) as either “positive”, “negative” or “unclear”, in case an item was inadequately reported upon. An unweighted Cohen’s kappa (k) coefficient was calculated to quantify the agreement between the reviewers. Agreement was scored as poor (0.0), slight (0.0 to 0.2), fair (0.21 to 0.4), moderate (0.41 to 0.6), substantial (0.61 to 0.8), to almost perfect (0.81 to 1.0) [14].

### Data analysis

Two reviewers performed the data extraction independently. In case data were lacking or not clearly described in the original paper the authors were approached to supply the raw data. In an attempt to increase readability, we summarized the values of the data on PPT and synchronized the two frequently applied PPT scores (kg/cm and kPa) into kPa by using a converter application, applying the following formula: 1 Kilogram-force/Square Centimeter (kg/cm^2^) = 98.0665 Kilopascal (kPa).

We summarized all mean values (kPa) of muscle sites found in each of the selected studies. The mean values were calculated separately for adults and gender. In case pooling of data was considered we assessed the following sources: classification of headache, age, site of measurement, and measurement units. We pooled data using the random effect model and the RevMan software program, version 5.3 [15].

## Results

### Flow of studies through the review

The flow diagram of the study selection is presented in Fig. 1. We searched publications on selected electronic databases (PubMed, Cinahl, and Embase.com) published during the period January 2004–August 2015. It was not necessary to consult a third reviewer for the selection and inclusion of studies. The studies that met the inclusion criteria were assessed for risk of bias.

Differences in score between both reviewers were discussed and solved. Arbitration was not needed. The score on risk of bias between both reviewers showed an overall agreement of 92% and had an unweighted Kappa of 0.6 (substantial agreement) (Table 1). Most important items and outcome of the studies are summarized in Table 2.

**Table 1:** Risk of bias assessment

Study	Definition of patient group	Definition of control group	Selection bias	Blinding of outcome assessment	Identifying of confounders
Ashina, 2005 [25]	Y	Y	Y	N	Y
Chua, 2011 [31]	Y	Y	Y	Y	Y
Engstrom, 2013a [16]	Y	Y	Y	Y	Y
Engstrom, 2013b [17]	Y	Y	Y	Y	Y
Engstrom, 2014a [18]	Y	Y	Y	Y	Y
Engstrom, 2014b [26]	Y	Y	Y	Y	Y
Fernández-de-Las-Peñas, 2007a [27]	Y	Y	Y	Y	Y
Fernández-de-Las-Peñas, 2007b [28]	Y	Y	Y	Y	Y
Fernandez-De-Las-Penas, 2008 [19]	Y	Y	Y	Y	Y
Fernandez-De-Las-Penas, 2010 [20]	Y	Y	Y	Y	Y
Filatova, 2008 [6]	Y	Y	Y	N	Y
Florencio, 2015 [21]	Y	Y	Y	Y	Y
Grossi, 2011 [22]	Y	?	Y	Y	Y
Peddireddy, 2009 [29]	Y	Y	Y	Y	Y
Tüzün, 2005 [30]	Y	Y	Y	N	Y
Uthaikhup, 2009 [23]	Y	Y	Y	N	Y
Zito, 2006 [24]	Y	Y	Y	Y	Y

Score: Y = information is adequate, ? = information is unclear, N = information is absent

**Table 2:** Study characteristics

Author/ year of publica-tion	Population				IHS criteria/Sjaastad	Blin-ding Y/N	Device and outcome			PPT sites		Reported confounders	Outcome: PPT values kPa (sd) in headache versus controls	
	Headache type	Control, matching,Setting	Age: mean (sd)	F/M			Electronic/ mechanical	Probe size	Out-come	Relevant cervical site	Extra cephalic site			
Engstrom [17] Norway	Migraine *n* = 33	*n* = 34 Open population	Migraine: sleep-related 39.4 (14.3) Not sleep- related 33.9 (11.4 Controls: 39.6 (13.7)	F/M	ICHD II	Y	Electronic algometer type II, Somedic Sales AB, Sweden	1 cm2	kPa/s	trapezius muscle, splenius muscle	distal phalanx middle finger	gender, age,interictal, medication	Average mean PPT score of m. splenius, m. trapezius, M. temporalis, and distal phalanx middle finger . Sleep- related migraine: 586 (141) *p* < 0.05 Not sleep-related migraine: (519) (125) *p* < 0.05 Controls: 661 (249)	
Engstrom [16] Norway	Migraine *n* = 50	*n* = 34 Open population	Migraine: interictal: 36.4 (12.9) pre-ictal 41.7 (11.3) post-ictal 41.1 (11.2) Controls: 39.6 (13.7)	F/M	ICHD II	Y	Electronic algometer type II,Somedic Sales AB, Sweden.	1 cm2	kPa/s	trapezius muscle, splenius muscle	distal phalanx middle finger	gender, age, fibromyalgia, ictal, medication	Average mean PPT score of m. splenius, m. trapezius, m. temporalis, and distal phalanx middle finger Interictal migraine: 549 (135) NS Pre-ictal migraine: 582 (194) NS Post-ictal migraine: 539 (70) NS Controls 661 (249)	
Engstrom [18] Norway	Migraine *n* = 53 ETTH *n* = 20	*n* = 34 Matched on age and gender, Open population	M: 38.2 (12.0) ETTH 40.9(13.5) Controls M: 39.6 (13.7) Controls ETTH 41.2 (13.6)	F/M	ICHD II	Y	Electronic Algometer type II, Somedic Sales AB, Sweden	1 cm2	kPa/s	trapezius muscle, splenius muscle	distal phalanx middle finger	gender, age, interictal, medication	Average mean PPT score of m. splenius, m. trapezius, m. temporalis, and distal phalanx middle finger Total Migraine (*n* = 33) 549 (135) NS Total TTH (*n* = 20) 543 (191) NS Sleep migraine (*n* = 15): 586 (141) NS CTTH (n-8) 506 (215) *p* < 0.05 Non sleep migraine (*n* = 18): 519 (125) *p* < 0.05 ETTH (*n*-12) 598 (144) NS Controls (*n* = 34) 661 (249)	
Fernandez de las Penas [19] Spain	Unilateral Migraine *n* = 25	*n* = 25 Hospital	Migraine: 32 (7) Controls: 31 (9)	F/M	ICHD II	Y	A pressure algometer connected to a sensor recording pressure	1 cm2	kg/cm2	trapezius muscle		gender, age, interictal, medication	Trapezius muscle Migraine; Female Symptomatic side 137.3 (39.2) *p* < 0.001 Non Symptomatic side 176.5 (39.2 *p* < 0.01 Controls: Symptomatic side 235.4 (39.2) Non-symptomatic side 235.4 (49) Migraine,; Male Symptomatic side 186.3 (58.8) *p* < 0.001 Non symptomatic side235.4 (58.8) *p* < 0.01 Controls Symptomatic side 264.8 (39.2) Non symptomatic side274.6 (39.2)	
Fernandez de las Penas [20] Spain	Unilateral Migraine *n* = 20 CTTH *n* = 20	*n* = 20 Matched on age and gender, Hospital	Unilateral migraine: 37.9 CTTH: 39. 8 Controls: 37.8	F	ICHD II	Y	Electronic algometer (Somedico type 2, Sollentuna, Sweden)	1 cm2	kPa/s	trapezius muscle, sub-occipital insertions, levator scapula muscle		gender, age, interictal, medication, fibromyalgia/WAD	Trapezius muscle CTTH Left: 246.3 (58.5) *p* < 0.001 Right: 257.5 (56.9) *p* < 0.001 Migraine Symptomatic side 276.1 (53.4)*p* < 0.001 Non-symptomatic side287.1 (84.3)*p* < 0.001 Controls Left:337.5 (33.1) Right: 338.3 (29.9)	Suboccipital region =suboccipital insertions CTTH Left 164.6 (33.1) *p* < 0.001 Right 172.3 (38.5) *p* < 0.001 Migraine Symptomatic 240.3 (50.5) *p* > 0.001 Non-symptomatic side 253.5 (54.1) *p* < 0.001 Controls Left 333.2 (42.8) Right 344.3 (41.4)
Filatova [6] Russia	chronic migraine *n* = 25 CTTH n = 25 Mixed headache *n* = 19	*n* = 18 Headache Center	CTTH+ chronic migraine + mixed headache :40.1 (12.3) Controls: 43.1(18.1)	F/M	ICHD II	N	Hand-held pressure algometer (Commander Algo-meterTM, JTech)	not repor- ted	lb/cm	trapezius muscle, C2		gender, age, ictal, medication, fibromyalgia/WAD	Average mean PPT score of forehead V1, temple, parietal area, posterior neck/C2, shoulder/trapezius CTTH + Chronic migraine): 284.4 (88.3) *p* < 0.005 Controls: 362.8 (117.7)	
Florencio [21] Brazil	Migraine *n* = 30	*n* = 30 Matched on gender (females) Headache Center	Migraine: 37 Controls: 32	F	ICHD III	Y	Digital manual dynamo-meter(DDK-10 Kratos)	1 cm2	kg/cm2	trapezius muscle, SCM muscle, sub-occipital muscles, levator scapula muscle, scaleni muscle		gender, age, interictal, medication, fibromyalgia/WAD	Trapezius muscle Migraine: Both sides 245.2 (9.8) *p* = 0.046 Controls Both sides 274.6 (19.6)	Suboccipital region =suboccipital muscles Migraine Both sides: 156.9 (9.8) *p* < 0.05 Controls Both sides: 235.4 (9.8)
Grossi [22] Brazil	Episodic migraine *n* = 15 Chronic migraine *n* = 14	*n* = 15 Headache Center	Episodic migraine: 36.3 (10.3) Chronic Migraine: 38.0 (10.4) controls: 39.9 (10.5)	F	ICHD II	Y	Digital manual dynamometer (DDK-10, Kratos)	1 cm2	kg/cm2	trapezius muscle, trapezius insertion SCM,		gender, age, medication, fibromyalgia/WAD	Trapezius muscle Episodic migraine Left 250 (91.2) *p* < 0.05 Right 263.8 (99)) *p* < 0.05 Chronic migraine Left 260.9 (82.4) *p* < 0.05 Right 290.3 (93.2) NS Controls Left 325.6 (95.1) Right 342.3 (81.4)	Suboccipital region (=trapezius insertion) Episodic migraine Left 224.6 (68.7) NS Right 222.6 (92.2) NS Chronic migraine Left 241.2 (79.4)NS Right 257.9 (85.3)NS Controls Left283.4 (72.6) Right285.4 (87.3)
Uthaikhup [23] Australia	Migraine *n* = 26 ETTH *n* = 10 CeH *n* = 24 unclassifiable*n* = 33	*n* = 44 Open population and University Community	Migraine: 66.6 (4.8) ETTH: 65.3 (4.5) CeH: 65.4 (4.7) Unclassifiable: 66.2 (4.4) controls: 66.4 (4.1)	F/M	ICHD II and Sjaastad	not reported	Electronic algometer (Somedic AB, Farsta, Sweden)	1 cm2	kPa/s	bilaterally over the articular pillars of the cervical segment C2–3	tibialis anterior	gender, age, medication, fibromyalgia	The articular pillars of the cervical segment C2–3 Migraine: ETTH: 238.2 (104) NS180.7 (85) NS CeH: Unclassifiable headache: 242.4 (105.0)NS 210.6 (113.9) NS Controls: 243.2 (105.9)	
Zito [24]. Australia	Migraine *n* = 25 CeH *n* = 27	*n* = 25 Hospital, GP, PT and controls in open population	Migraine: 22.9 (3.5) CeH: 25.3 (3.9) Controls: 22.9(3.5),	F	ICHD II and Sjaastad	Y	Pressure algometer (PD&T—Italy)	1 cm2	kg/cm2	C2 the trans-verse process of C4 and the C2/3 zygapophyseal joint		gender, age, interictal	C2 GONTransverse process C4C2/3 zygapoph. Joint Migraine:294.2 (107.9) NS441.3 (196.1) NS 372.7 (117.7) *p* < 0.05323.6 (117.7) NS CeH:323.6 (107.9) NS 421.7 (137.3) NS 382.5 (107.9) *p* < 0.05333.4 (98.1) NS Controls: 313.8 (98.1) 480.5 (156.9) NS 441.3 (117.7) 353 (107.9)	
Ashina [25] Denmark	CTTH *n* = 20	*n* = 20 Matched on age, gender, body weight and height, blood pressure Hospital	CTTH: 46 (11) (26–63) Controls: 46 (12) (26–62)	F/M	ICHD II	N	Electronic algometer Somedic AB, Horby, Sweden	0.5 cm2	kPa/s.	trapezius muscle	tibialis anterior	gender, age, ictal, medication	Trapezius muscle. CTTH: 390 (39) *P* < 0.05 Controls: 519 (38)	
Engstrom [26] Norway	CTTH *n* = 12 ETTH *n* = 8	*n* = 20 Matched on age and gender Open population	TTH: 41.2 (13.6) Controls: 40.9 (13.5)	F/M	ICHD II	Y	Electronic Algometer type II, Somedic Sales AB, Sweden	1 cm2	kPa/s	trapezius muscle, splenius muscle	distal phalanx middle finger	gender, age, ictal, medication, fibromyalgia	Average mean PPT score of m. splenius, m. trapezius, m. temporalis, and distal phalanx middle finger CTTH: 506 (215) *p* < 0.05 ETTH: 598 (144) NS Controls 678 (251)	
Fernandez de las Penas [28] Spain	CTTH *n* = 20	*n* = 20 Matched on age and gender Hospital	CTTH: 36 (11)), controls: 35 (9)	F/M	ICHD II	Y	Mechanical pressure algometer	1 cm2	kg/cm2.	trapezius muscle		gender, age, ictal, medication	Trapezius muscle CTTH Left 147.1 (39.2) Right 137.3 (39.2) Controls Left 245.2 (49) *p* < 0.001 Right 255 (49) *p* < 0.001	
Fernandez de las Penas [27] Spain	CTTH *n* = 25	*n* = 25 Hospital	CTTH: 41 (14) Controls 39 (13)	F/M	ICHD II	Y	Mechanical pressure algometer	1 cm2	kg/cm2	trapezius muscle		gender, age, ictal, medication	Trapezius muscle CTTH Left 147.1 (49) *p* < 0.001 Right 142.2 (49) *p* < 0.001 Controls Left 235.4 (49) Right 254.2 (39.2)	
Peddireddy [29] Denmark	CTTH *n* = 30	n = 30 Matched on age and gender Open Population	CTTH male 48.4 (7.9) female 44.8 (3.2) Controls: male 50.8 (1.4) and female 44.8 (2.2)	F/M	ICHD II	Y	Electronic algometer (Somedic AB, Stockholm, Sweden)	1 cm2	kPa/s	splenius capitis muscle		gender, age, ictal, medication	No exact PPT retrievable from text, figures or tables. The PPT of scalenus muscle did not significantly differ between CTTH and controls	
Tüzün [30] Turkey	CTTH *n* = 35	*n* = 70 Matched on gender University students	CTTH: 18.9 (1.2) Controls: 19.2 (1.7)	F/M	ICHD II	N	Handheld algometer (Pain Diagnostic and Thermo-graphy,USA)	1 cm2	kg/cm2	trapezius muscle, sub-occipital muscles		gender, age, interictal, medication	Trapezius muscle CTTH: left: 470.7 (166.7) *p* = 0.002 Right: 529.5 (176.5) *p* < 0.001 Controls: Left 666.8 (284.3) Right 686.4 (284.3)	Suboccipital region =suboccipital muscles CTTH: Left 264.8 (68.7) *p* < 0.001 Right 294.2 (88.3) *p* < 0.0001 Controls: Left 353 (112.8) Right 372.7 (107.9)
Chua [31] Nether-lands	CeH *n* = 17 Non CeH *n* = 10	*n* = 27 Matched on age and gender Hospital	CeH: 50.6 (11.1) Non CeH:54.5 (7.9) Pain free:52.1 (10.4)	F/M	Sjaastad	Y	Electronic algometer Somedic Production AB, Farsta, Sweden	1 cm2	kPa/s	sterno-cleido- mastoideas greater occipital nerve, C4–5 zygopo-physisial joint		gender, age fibromyalgie, medication	Average mean PPT score of sterno-cleido- mastoideas, greater occipital nerve, and C4–5 zygopo-physisial joint CeH painful side 935 (256) non painful side 937 (332) *p* < 0.001 Non CeH painful side 1109 (465) non painful side 1114 (522) *p* < 0.001 Controls 497 (215)	

*PPT* pressure pain threshold, *IHS* International Headache Society, *ETTH* episodic tension-type headache, *CTTH* chronic tension-type headache, *CeH* cervicogenic headache, *NS* not significant, *p* > 0.05

### Characteristics of studies

All 17 selected studies are case-control studies. A total of 671 participants with headache (episodic TTH *n* = 38, chronic TTH *n* = 187, migraine *n* = 316, CeH *n* = 68, unclassifiable headache *n* = 62) and 491healthy controls were analyzed.

PPTs were assessed using electronic or mechanical devices at specific sites in the cranio-cervical region. Most investigated types of headache were CTTH and migraine. The majority of PPT values in the cranio-cervical region are expressed as separate scores for each crane-cervical muscle, joint or transverse process. The most investigated and best recorded site among the different headache disorders was the midpoint between vertebrae C7 and acromion in the trapezius muscle.

Only the upper trapezius muscle fulfilled the aforementioned required criteria for homogeneity. Pooling of results from other sites was not possible due to variations in localization or definition of sites, and lack of specified PPT scores.

### Included studies

#### Migraine versus controls

We retrieved ten studies that described PPT in migraine [6, 16–24].

In the trapezius muscle, four studies [19–22] found significantly lower mean PPT values in participants with migraine compared to controls with a pooled mean difference of (kPa) -55.75 [95%CI -79.80, −31.70] (Table 3).

**Table 3:**
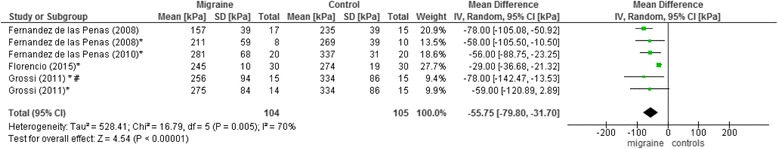
Results of meta-analysis of pressure pain thresholds (kPa) of trapezius muscle (midpoint between vertebrae C7 and acromion) in migraine versus control, * results of pressure pain thresholds in females, # episodic migraine

In the suboccipital region (suboccipital insertion, trapezius insertion, suboccipital muscles) three studies [20–22] described significantly lower PPT values compared to controls. This result contrasts with that of Zito et al. who found no significant difference [24] (Table 2).

Mean PPT scores of a combination of measurements on the splenius muscle, trapezius muscle, temporalis muscle, and index finger in migraine were described in two studies by Engstrom et al. in which one study reported significant lower values between not sleep-related migraine versus controls (kPa: 519, sd 125 vs kPa: 661, sd 249 *p* = 0.05, 16,18).

#### TTH versus controls

Ten studies [6, 18, 20, 23, 25–30] reported PPT values in the cranio-cervical region in participants with TTH. In the trapezius muscle, five studies [20, 25, 27, 28, 30] found significant lower PPT values in participants with chronic TTH compared to controls with a pooled mean difference of (kPa) -109.57 [95%CI -129.25, −89.88] (Table 4).

**Table 4:**
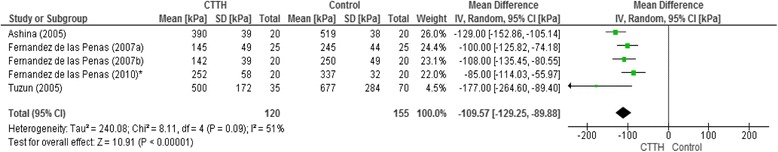
Results of meta-analysis pressure pain thresholds (kPa) of trapezius muscle (midpoint between vertebrae C7 and acromion) in chronic tension-type headache (CTTH) versus control, * results of pressure pain thresholds in females

At the left and right suboccipital region (suboccipital insertion, suboccipital muscles) the PPT were significant lower in participants with chronic TTH versus controls in two studies (*p* < 0.002) [20, 30]. Mean PPT scores of a combination of measurements on the splenius muscle, trapezius muscle, temporalis muscle, and index finger were significant lower in chronic TTH patients (*p* < 0.05), while no significant were observed in episodic TTH compared to controls [18, 26]. One study showed significantly lower PPT values (*p* > 0.05) in the splenius capitis muscle in chronic TTH patients [29]. In elderly patients with episodic TTH, no significant differences in PPT scores at the upper neck were detected [23].

#### CeH versus controls

Three studies reported PPT values in the cranio-cervical region in participants with CeH. Zito et al. found no between-group differences in PPT scores at the C2–3 zygapophyseal joint, but significantly lower PPTs in the area over the transverse process of C4 in comparison to the control group (P 0.05) [24]. No significant difference in PPT score over the articular pillars of the cervical segment C2–3 was reported between elderly participants (65.4 years, sd 4.7) [23].

One study described PPT in the pain-free reference area (i) in the thigh; (ii) superior insertion of sternocleidomastoid; (iii) temporalis muscle and (iv) ophthalmic division of the trigeminal and showed a significant difference (F-5.63, *p* < 0.001) between CeH group and participants with only neck pain using a multivariate general linear model and a significant site effect compared to controls (F -17.39, *p* < 0.001, 31).

#### Between headache groups

Participants with chronic TTH show significant lower PPT values at 3 different sites in the neck region (i. the suboccipital muscle insertions, ii. transverse process of C5, iii. Middle point between the spinous process of C7 and the acromion) compared to participants with strictly unilateral migraine [20]. Filatova et al. found no significant difference in average mean PPT scores of the forehead, temple and neck (trapezius muscle and C2 point) between chronic TTH and chronic migraine [6].

Engstrom et al. reported significant lower values (*p* < 0.05) on average mean PPT score from splenius muscle and trapezius muscle in chronic TTH versus episodic TTH [26]. There were no significant differences of these sites between interictal (kPa: 549, sd 135), pre-ictal (kPa: 582, sd 194), and post-ictal migraine (kPa: 539, sd 70) [17]. Between participants with episodic and chronic migraine also no significant differences in PPT were detected in the trapezius muscle and sternocleidomastoideus muscle [22]. One study found no significant differences in PPT at the transverse process of C4 and the C2/3 zygapophyseal joint between CeH and migraine [24].

In elderly participants with episodic TTH, migraine, CeH, or unclassifiable headaches, no significant differences are described in PPT in the neck region (bilaterally over the articular pillars of the cervical segment C2–3) [23].

### Gender differences

Three studies analyzed differences in PPT values between adult male and female participants and reported lower PPT values in neck points in females [19, 27, 29]. In two studies, these differences reached statistical significance. Females with chronic TTH showed significantly lower PPT values in the splenius capitis muscle (*P* < 0.05) [29]. Females with migraine and in the healthy control group reported lower PPT levels (*P* < 0.001) in the upper trapezius muscle than males [19].

### Association between PPT values and headache parameters

#### PPT values and frequency of headache

In total eight studies reported the association between PPT values of several cranio-cervical sites and headache frequency in chronic TTH [6, 20, 23, 27, 29, 30] and migraine [6, 19–21, 23]. Fernandez de las Penas et al. showed a significant negative association in migraine and chronic TTH between PPT of trapezius muscle and frequency of headache, whereas other studies [6, 19–21, 23, 27, 29, 30] did not find a significant association (Table 2).

### PPT values and intensity of headache

Significant negative association (*P* < 0.05) between PPT values in the cervical region and headache pain intensity was observed in two studies: one paper, including participants with chronic TTH [28] and another describing participants with migraine [20]. Most studies reported no significant association between PPT values and headache intensity in chronic TTH [6, 23, 27, 29, 30] or migraine [6, 21, 23].

### PPT values and duration of headache

In chronic TTH two studies [20, 28] reported a significant relation between PPT and duration of headache, whereas four studies [23, 27, 29, 30] found no significant association. In migraine, one study observed a significant negative association between PPT and duration over 3 points in the neck region (*P* > 0.05) [20]. Three studies [19, 21, 23] reported no significant relation between duration of migraine and PPT in the cranio-cervical region.

## Discussion

This systematic review provides PPT value ranges over the cranio-cervical region of healthy controls, migraine, TTH and CeH. This is of importance given the growing interest of prophylactic treatments aimed at the cranio-cervical region.

Measurement of PPT has played an important role in elucidating the pathophysiology of chronic headache. By describing PPT values over the cranio-cervical region this review contributes to the understanding of pathophysiological mechanism in different types of headache.

We were able to include 17 papers with low risk of bias. We found reduced PPT in the cranio-cervical region primarily in migraine and chronic TTH compared to asymptomatic controls. Comparison among headache types indicates that reduced PPT are primarily a feature of chronic TTH. This is in line with a narrative review that summarized the literature on PPT measurement in TTH till 2010 [4].

The most frequently recorded PPT measurement was located at the midpoint between vertebrae C7 and acromion in the trapezius muscle. Here, pooling of data was allowed based on identical headache diagnosis (ICHD II, ICHD III, Sjaastad criteria), age, site of measurement, and outcome of measurement. Subsequently, the mean PPT values were calculated and showed a significant mean difference in participants with chronic TTH versus controls as well in participants with migraine versus controls.

The suboccipital region was the second most reported site of measurement and includes the suboccipital muscles insertion, trapezius muscle insertion, and suboccipital muscles. Pooling of data was not feasible due to heterogeneity of measured sites in this region. However, it is noteworthy that in four out of five studies the suboccipital region reported significantly lower PPT in chronic TTH and migraine. These significantly lower PPT thresholds in the trapezius muscle and suboccipital sites reflect altered pain perception in this regions and support the pathophysiological model of sensitization in migraine and chronic TTH [32]. These findings highlight the importance of PPT assessment at aforementioned cranio-cervical sites in headache research and evaluation of treatment.

In all studies PPT are generally lower in females than in males. This is a consistent finding and is in line with other studies [9, 33].

Most studies found no significant association of headache characteristics (frequency, intensity, duration) with PPT values within the different types of headache. This can be due to the combination of small sample sizes and the wide range of PPT values.

One of the strengths of this review is that we used state of the art methods for performing systematic reviews and rigorously applied these guidelines for study selection (e.g. by using an independent librarian to compose and execute the search strategy), screening for eligibility, assessment of risk of bias, and analysis of data. PPT may differ between types of headache and within TTH [4]. Consequently, we regarded the classification of headache, especially TTH, as an essential and key element in the assessment of studies. Therefore, we limited our search to studies that were published after 2004, the year of publication of the ICHD II, since this classification provided an updated and more detailed classification of TTH. At the same time, this limitation can be considered as a weakness of this study because this paper covers not all available evidence on PPT measurement over the cranio-cervical region.

Another strength of our study is that we were able to pool data at one point that was carefully selected based on descriptions in the protocols and representations of the figures.

For the measurement of PPT different instruments are used. The validity of the reported results on PPT values in the selected studies therefore depends partly on the reliability of measurement. Some studies have assessed the intra- and inter-examiner reliability and reported high reliability scores (range ICC: 0.82–0.99) of the PPT measurement for both mechanical and electronic devices [18, 24, 27, 28]. These findings are in line with previous research [2, 34, 35]. Given these high reliability scores, we are confident that the results of this review are not hampered by insufficient reliability of the applied measurement methods.

Although the performance of the PPT measurement was comparable across the studies there was a great variety of sites measured in the cranio-cervical region. Some studies reported sum scores, i.e. average mean scores of the PPT of multiple sites in the cranio-cervical region or cranio-cervical sites in combination with extra-cephalic sites. Besides these differences in sites also the anatomical description of sites was not accurate with the exception of the midpoint in the trapezius muscle between vertebrae C7 and acromion. These methodological shortcomings hamper the reproduction of the study and generalizability of results. Therefore, we would recommend the standardization of measurements on well-defined spots and reporting of separate scores on sites in future studies. This will allow future pooling of data which in turn will lead to more solid and robust conclusions.

We would further recommend clinicians and future researchers to analyze the results of male and female participants separately.

At present, no grading system is available to rate epidemiological associations. Even though, in regard to the results of this systematic review, we want to consider the following methodological aspects of the included studies. All studies had a case-control design and their risk of bias was assessed to be low. Although studies applied the ICHD II or III for the inclusion of participants, not all studies reported on the severity of headaches and consecutiveness of patients. The majority of the studies recruited participants with headache from hospitals or headache clinics and this may overestimate the results. We found that most studies included a sufficient number of patients and controls and reported mean PPT values with broad confidence intervals. One of the reasons of this broad CI around the mean is that there is a great variability of PPT scores between individuals. The intra- (stability of the measurement) and inter-rater reliability (differences between subjects) show to be good. Still, all studies show lower, but not in all studies significantly lower, PPT values in the cranio-cervical region in patients with headache versus controls. When we take the forementioned methodological aspects into account to estimate the body of evidence, we conclude that this review represents moderate evidence (level B) between PPT values in the cranio-cervical region and headache.

## Conclusion

We conclude that the PPT values of the trapezius muscle are significantly lower in migraine and chronic TTH compared to controls. In most studies, no significant associations were reported between PPT values in the cranio-cervical sites and headache characteristics such as frequency, duration or intensity. The increased sensitivity of cranio-cervical sites supports the neurophysiological model of sensitization in migraine and chronic TTH. Therefore, measuring PPT in the cranio-cervical region is a valuable tool for clinicians and researchers.

## Additional file

Additional file 1: Appendix 1. Search strategy.
