# Supplementary material for: Pressure pain thresholds over the cranio-cervical region in headache: a systematic review and meta-analysis
Source: J Headache Pain. 2018 Jan 26;19(1):9. doi: 10.1186/s10194-018-0833-7 (PMC5786597; doi:10.1186/s10194-018-0833-7)
Supplement: Additional file 1: Appendix 1. — Search strategy. [file 10194_2018_833_MOESM1_ESM.docx]

Appendix 1. Search strategy (August 2015)

**Pubmed**

"Headache Disorders"[Mesh:NoExp] OR "Headache Disorders, Primary"[Mesh:NoExp] OR "Migraine Disorders"[Mesh:NoExp] OR "Migraine with Aura"[Mesh] OR "Migraine without Aura"[Mesh] OR "Ophthalmoplegic Migraine"[Mesh] OR "Tension-Type Headache"[Mesh] OR "Post-Traumatic Headache"[Mesh] OR cervicogenic headache*[tiab] OR post traumatic headache*[tiab] OR posttraumatic headache*[tiab] OR cgh*[tiab] OR ceh*[tiab] OR tth*[tiab] OR ctth*[tiab] OR migrain*[tiab] OR tension type headache*[tiab] OR tensiontype headache*[tiab] OR nummular headache*[tiab] OR cervicogenic headache*[ot] OR post traumatic headache*[ot] OR posttraumatic headache*[ot] OR cgh*[ot] OR ceh*[ot] OR tth*[ot] OR ctth*[ot] OR migrain*[ot] OR tension type headache*[ot] OR tensiontype headache*[ot] OR nummular headache*[ot]

"Pain Threshold"[Mesh] OR ppt*[tiab] OR pain threshold*[tiab] OR algomet*[tiab] OR ppt*[ot] OR pain threshold*[ot] OR algomet*[ot]

**Embase**

**'chronic daily headache'**/exp OR **'migraine'**/exp OR **'primary headache'**/exp OR **'secondary headache'**/exp OR **'tension headache'**/exp OR (**cervicogenic** NEAR/4 **headache***):ab,ti OR (**'post traumatic'** NEAR/4 **headache***):ab,ti OR (**posttraumatic** NEAR/4 **headache***):ab,ti OR **cgh***:ab,ti OR **ceh***:ab,ti OR **tth***:ab,ti OR **ctth***:ab,ti OR **migrain***:ab,ti OR (**'tension type'** NEAR/4 **headache***):ab,ti OR (**tensiontype** NEAR/4 **headache***):ab,ti OR (**nummular** NEAR/4 **headache***):ab,ti

**'pain threshold'**/de OR **'pressure pain threshold'**/exp OR **ppt***:ab,ti OR (**pain** NEAR/4 **threshold***):ab,ti OR **algomet***:ab,ti

**Cinahl**

TI ( “cervicogenic headache*” OR “post traumatic headache*” OR “posttraumatic headache*” OR cgh* OR ceh* OR tth* OR ctth* OR migrain* OR “tension type headache*” OR “tensiontype headache*” OR “nummular headache*” ) OR AB ( “cervicogenic headache*” OR “post traumatic headache*” OR “posttraumatic headache*” OR cgh* OR ceh* OR tth* OR ctth* OR migrain* OR “tension type headache*” OR “tensiontype headache*” OR “nummular headache*” )

TI ( ppt* OR “pain threshold*” OR algomet* ) OR AB ( ppt* OR “pain threshold*” OR algomet* )
